# Editorial: Improving the gut microbiome: applications of fecal transplantation in disease

**DOI:** 10.3389/fmed.2023.1203448

**Published:** 2023-05-19

**Authors:** Sandra García-Mateo, Angel Lanas

**Affiliations:** ^1^Service of Digestive Diseases, University Clinic Hospital Lozano Blesa, Zaragoza, Spain; ^2^Instituto de Investigación Sanitaria de Aragón (IIS Aragón), Zaragoza, Spain; ^3^University of Zaragoza, Zaragoza, Spain; ^4^Centro de Investigación Biomédica en Red de Enfermedades Hepáticas y Digestivas (CIBERehd), Madrid, Spain

**Keywords:** fecal transplant, bacteria, microbioma, gastrointestinal, inflammatory bowel conditions

“*Improving the gut microbiome: applications of fecal transplantation in disease*” is a Research Topic with the aim of point out the advances in gut microbiome as a therapeutic tool, not only for gastrointestinal disorders, but also for non-communicable diseases. As such, this editorial focuses on recent advances in fecal microbiota transplantation (FMT) in a wide spectrum of diseases highlighting both the windfalls and challenges of this promising therapeutic weapon as reflect the manuscripts submitted and published as part of this novel topic.

The progressive worsening of global obesity pandemic which predispose individuals to metabolic syndrome or cardiovascular disease in parallel with a severe downturn of lifestyle and diet despite the development of new, more personalized, and powerful drugs, has driven researchers to focus on new therapeutic targets. In line with this, in recent decades has emerged a more holistic paradigm of diseases. It assigns not only to microbiota, which englobe bacteria, virus, or protozoa, but also to microbioma, which encompasses also other molecules resulting of the interaction of microbiota and the immune system of the host, a principal role in the physiopathology of illness, being dysbiosis the new goal of the modern therapeutic approaches.

Microbiota-based therapies have been investigated since the 1950s as a treatment of gut dysbiosis to return metabolite levels and profiles to a healthy state as a result of a normal host enzymatic activity. Over the last decades, fecal microbiota transplantation (FMT) has become a potential treatment strategy by restoring a balanced microbiome to the host. However, recurrent *Clostridioides difficile* infection is currently the only indication of FMT as Chopra et al. have widely reported, despite their promising results in other non-communicable diseases.

Several clinical studies have been conducted using FMT in the setting of metabolic syndrome, obesity, and non-alcoholic fatty liver disease, not only in rats but also in humans as Liptak et al. describe in their review. In those studies, patients showed decreased gut microbial diversity, and after FMT from lean and healthy donors, an increase in microbial diversity, butyrate producing microorganisms, insulin sensitivity or improvement in liver necrosis were observed. Hopeful results were also noticed in hepatic encephalopathy, autism disorder, depression or Parkinson's disease, where improvements in behavioral and depressive symptoms, as well as constipation and other gastrointestinal symptoms were pointed out. Moreover, FMT not only plays a significant role among non-communicable or neurological diseases, but also surprisingly in others such as graft-vs-host disease or HIV infection. Ouyang et al. thoroughly reviewed the evidence and highlighted the importance of clarifying some areas of uncertainty in these specific diseases claiming more clinical trials, a statement that we had not overlook either.

However, even when the results reported were positive, most of these studies had some common weaknesses: FMT impact was only evaluated after a short time after the procedure, studies were scarce in some fields and had substantial methodological differences between them. All these shortcomings make hard to assume their conclusions (Figure 1).

**Figure 1:**
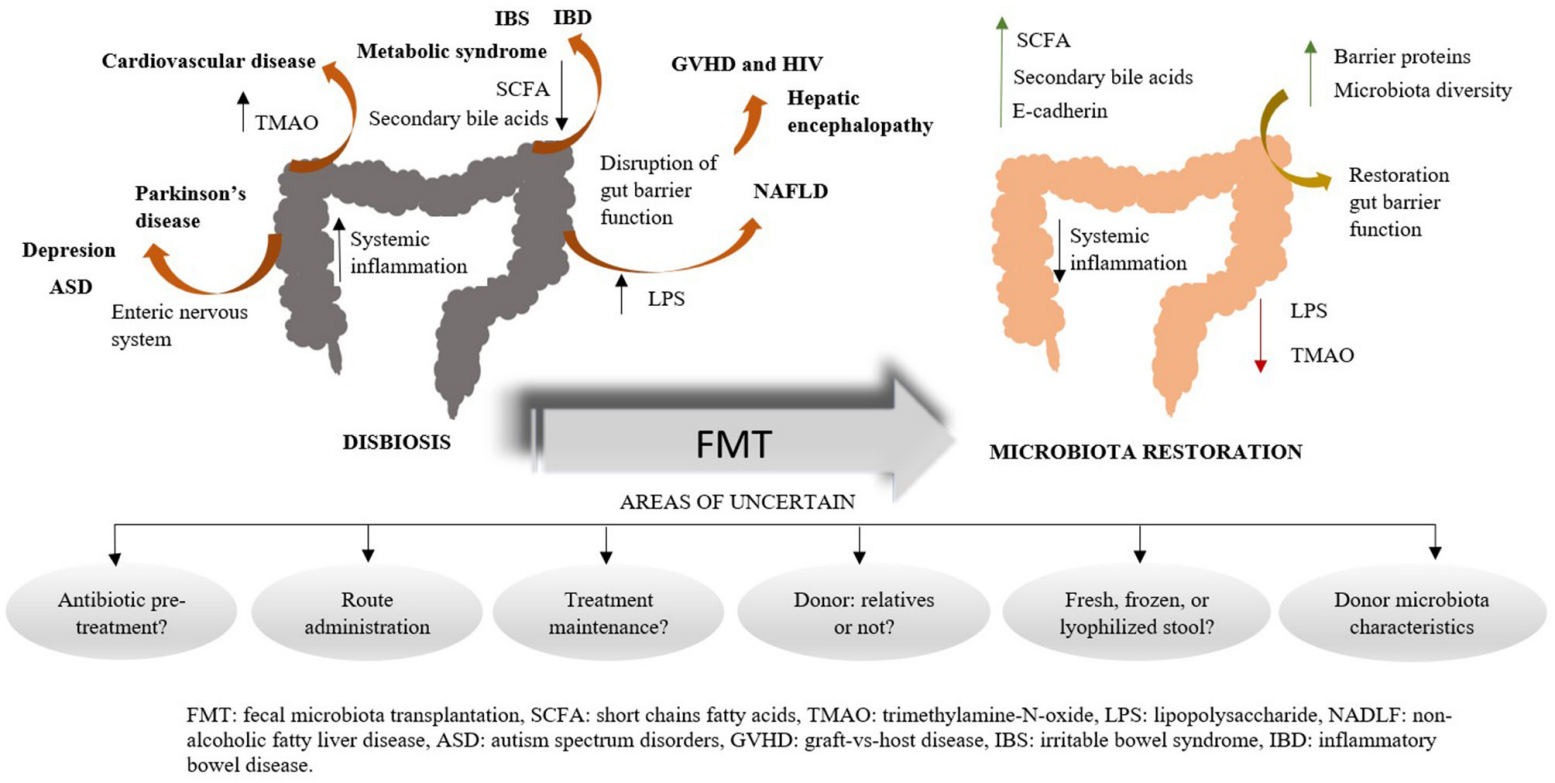
Fecal microbiota transplantation as a promising and developing tool for gastrointestinal and non-gastrointestinal diseases by microbiota restoration. FMT, fecal microbiota transplantation; SCFA, short chains fatty acids; TMAO, trimethylamine-N-oxide; LPS, lipopolysaccharide; NADLF, non-alcoholic fatty liver disease; ASD, autism spectrum disorders; GVHD, graft-vs-host disease; IBS, irritable bowel syndrome; IBD, inflammatory bowel disease.

To date, more robust evidence has been reported through several randomized controlled trials to demonstrate the efficacy of FMT in the setting of inflammatory bowel disease (IBD) (Zhang X. et al.). A systematic review of 30 studies carried out by Zhang J. et al. showed that the microbiome of FMT responders were similar to their donors, with an increase of short-chain fatty acids (SCFA) producing taxa. However, these results should be taken with care once again, because there were important differences among the results of the studies included in the systematic review, probably due to their methodological differences, especially in the ways of FMT delivery and the number of infusions.

Although colonoscopy seems to be the most effective mechanism of FMT administration, other delivery methods such as nasal tube, capsules, sigmoidoscopy, or retention enema are available as Hamman et al. reported. Tkach et al. performed a randomized clinical trial to demonstrate the efficacy and safety of FMT via colonoscopy in patients with mild-to-moderate ulcerative colitis (UC), with great outcomes for tolerability and safety, but with no differences in stool frequency (*p* = 0.583), fecal calprotectin and microbiota composition between FMT and the standard care group. It is important to note that due to severity of some diseases, standard therapy should be continued during the trials, so the role of FMT in the improvement of patient conditions is usually difficult to ascertain.

In Crohn's disease (CD), clinical trials with FMT are scarce, with clearly worse steroid-free clinical remission rates after FMT compared with the results observed in UC patients. These differences may be explained by the presence of extensive lesions in the small intestine in CD. In line with this, other complementary options apart from FMT, which mainly treats the colon microbioma, should be considered. Given that microbioma of small intestine participates in the pathogenesis of some diseases such as CD, intestinal fluid transplantation (HIFT) (Chen et al.) may have a crucial role. In fact, treating the whole microbiota and not only the one limited to the colon could be more effective than FMT alone, as has been reported by Chen et al.

One of the weaknesses in that field is the absence of standard protocols. Researchers determine different follow-up periods and commonly tend to perform short-term follow-up studies with small sample sizes. Although it makes studies more ease to compare, they loss strength because the lack of long-term data.

In this way, it is important to outline the retrospective study reported by Cui et al. in 227 patients with irritable bowel syndrome (IBS) who underwent FMT and were evaluated at different follow-up time points with a maximum of 60 months. The conclusion was that the treatment effect declined over time and that repeated and periodic FMT treatment can significantly guarantee the long-term efficacy of this therapy. It is clear, however, that more studies are needed to determine the frequency and number of FMT to obtain a successful and long-lasting therapeutic effect.

On another note, Cui et al. also compare the efficacy among different ways of delivery. They found significant differences in the efficacy rates after the administration of FMT capsules when compared to nasointestinal tube and colonoscopy administration. Although colonoscopy seems to be preferable (Hamamah et al.), oral capsule features an easy route to implement, with less side effects being a non-invasive method which leads to better medical compliance.

It is important to highlight the potential role of FMT as a promising therapeutic tool. However, it is necessary to draw attention to the fact that it may involve certain risks. One of the most side effects reported among different studies is the transference of unknown pathogenic microorganism to the host (Orr). Thereby it has been reported not only an increased risk for sepsis or death, but also the risks of developing diseases in the future such a colorectal cancer or metabolic syndrome. Without a doubt whatsoever, the whole microbiota is a complex entity with microbes that have never been fully characterized, and need much more research in the different fields being explored so far.

Orr emphasized the importance of a good donor selection, because seemingly healthy donors may not necessarily be appropriate donors for FMT. He highlights the importance of developing tools to identify and prioritize factors that best support a healthy gut biota among recipients, being crucial not only donor biota diversity but also patient's preparation with antibiotics, and an adequate diet without industrial or processed food. However, much more remains to be learned in that field.

In that line, a new list of endogenous microorganisms with potential benefits but still without a long history of clinical use called next-generation probiotics (NGP), are currently being investigated as the next step of traditional probiotics in order to mitigate those limitations and risk of FMT (Wortelboer et al.). NGP have shown promising results restoring the gut microbiota in *Clostridioides difficile infection* (Chopra et al.). Wortelboer et al. published the case of *Anaerobutyricum soehngenii*, a NGP which has demonstrated to improve insulin-resistance with hopeful perspectives in the field of metabolic syndrome and obesity in both *in vivo* and *in vitro* studies as well as in humans.

By and large, although the promising results that have been currently publishing, more controlled and personalized procedures are needed to improve the long term success after FMT an mitigate the potential side effects of the procedures.

## Author contributions

Both authors listed have conducted the research and investigation process as well as they have prepared and presented the work approved for publication.

